# A bi-planar coil system for nulling background magnetic fields in scalp mounted magnetoencephalography

**DOI:** 10.1016/j.neuroimage.2018.07.028

**Published:** 2018-11-01

**Authors:** Niall Holmes, James Leggett, Elena Boto, Gillian Roberts, Ryan M. Hill, Tim M. Tierney, Vishal Shah, Gareth R. Barnes, Matthew J. Brookes, Richard Bowtell

**Affiliations:** a Sir Peter Mansfield Imaging Centre, School of Physics and Astronomy, University of Nottingham, Nottingham, NG7 2RD, UK; b Wellcome Centre for Human Neuroimaging, Institute of Neurology, University College London, 12 Queen Square, London, WC1N 3AR, UK; c QuSpin Inc., 331 South 104th Street, Suite 130, Louisville, CO 80027, USA

## Abstract

Small, commercially-available Optically Pumped Magnetometers (OPMs) can be used to construct a wearable Magnetoencephalography (MEG) system that allows large head movements to be made during recording. The small dynamic range of these sensors however means that movement in the residual static magnetic field found inside typical Magnetically Shielded Rooms (MSRs) can saturate the sensor outputs, rendering the data unusable. This problem can be ameliorated by using a set of electromagnetic coils to attenuate the spatially-varying remnant field. Here, an array of bi-planar coils, which produce an open and accessible scanning environment, was designed and constructed. The coils were designed using a harmonic minimisation method previously used for gradient coil design in Magnetic Resonance Imaging (MRI). Six coils were constructed to null $B_{x}$, $B_{y}$ and $B_{z}$ as well as the three dominant field gradients $dB_{x}/dz$, $dB_{y}/dz$ and $dB_{z}/dz$. The coils produce homogeneous (within ±5%) fields or field gradients over a volume of 40 × 40 × 40 cm^3^. This volume is sufficient to contain an array of OPMs, mounted in a 3D-printed scanner-cast, during basic and natural movements. Automated control of the coils using reference sensor measurements allows reduction of the largest component of the static field ($B_{x}$) from 21.8 ± 0.2 nT to 0.47 ± 0.08 nT. The largest gradient ($dB_{x}/dz$) was reduced from 7.4 nT/m to 0.55 nT/m. High precision optical tracking allowed experiments involving controlled and measured head movements, which revealed that a rotation of the scanner-cast by ±34° and translation of ±9.7 cm of the OPMs in this field generated only a 1 nT magnetic field variation across the OPM array, when field nulling was applied. This variation could be further reduced to 0.04 nT by linear regression of field variations that were correlated with the measured motion parameters. To demonstrate the effectiveness of the bi-planar coil field cancellation system in a real MEG experiment, a novel measurement of retinotopy was investigated, where the stimulus remains fixed and head movements made by the subject shift the visual presentation to the lower left or right quadrants of the field of view. Left and right visual field stimulation produced the expected responses in the opposing hemisphere. This simple demonstration shows that the bi-planar coil system allows accurate OPM-MEG recordings to be made on an unrestrained subject.

## Highlights

• The design and use of bi-planar coils for magnetic field nulling is described.
• Field nulling allows large subject movements during onscalp MEG recordings.
• Optical tracking shows high quality data can be acquired during these movements.
• A novel measurement of retinotopy where the subject moves their head is shown.

## 1 Introduction

Magnetoencephalography (MEG) is a method for non-invasively mapping electrophysiological activity in the human brain (Cohen, 1968). It produces images of brain function with high spatiotemporal resolution by measuring the magnetic fields generated outside the head by neuronal currents in the brain. MEG presents a significant engineering challenge: the fields generated above the scalp are of the order of tens of femtotesla (fT), which is more than 10^9^ times smaller than the Earth's magnetic field and orders of magnitude smaller than other sources of magnetic interference (Hämäläinen et al., 1993). Current MEG systems employ Superconducting QUantum Interference Devices (SQUIDs) to measure the very small neuromagnetic fields, and must be housed inside a Magnetically Shielded Room (MSR) to reduce static and interference fields. Although the sensitivity of SQUIDs is almost unrivalled, they generally require cooling using liquid helium and so must be operated inside a dewar arrangement (Hämäläinen et al., 1993). The resulting 'one size fits all' helmet that is used in current MEG systems means that the sensors are not optimally positioned relative to the head, and also limits the amount of head movement that subjects can make during recordings. The consequently unnatural environment of current MEG scanners also does not allow easy application of naturalistic stimuli. Furthermore, it can pose problems in recording from subject groups, such as patients or infants, who find it hard to keep their heads still relative to the MEG sensors. Although several valuable approaches for compensating for head movement within the confines of the conventional MEG helmet have been developed (Nenonen et al., 2012; Taulu et al., 2005; Wehner et al., 2008), large gross motion (e.g. motion of the head away from the helmet) remains a significant problem due to loss of signal, which cannot be compensated in post processing.

**Fig. 1:**
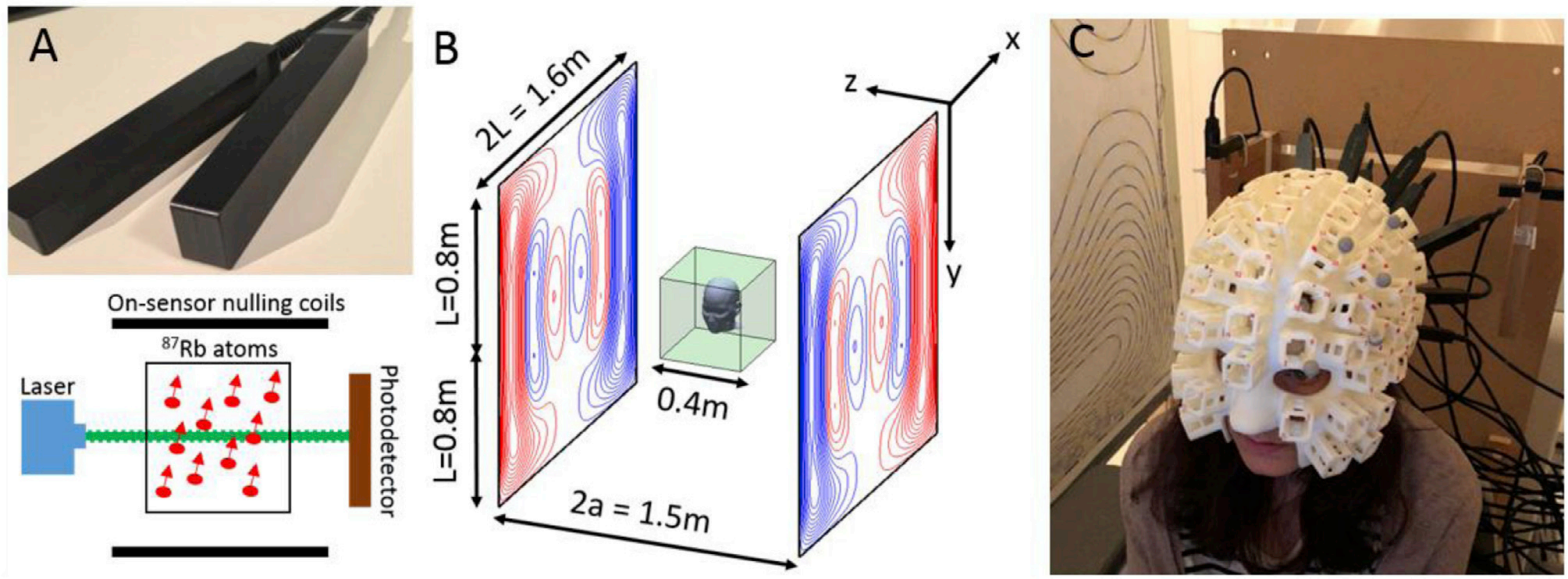
A) QuSpin OPMs are small, self-contained magnetic field sensors which operate by monitoring the transparency of a cell of rubidium-87 atoms. A circularly-polarised 795 nm laser spin-polarises the atoms. In the absence of an applied magnetic field the cell becomes transparent, while an applied field induces Larmor precession of the atoms which alters the transparency of the cell. The output of a photodetector then allows measurement of the local magnetic field. Three on-sensor coils are used to create a “zero-field” environment for the cell and to apply a modulation field perpendicular to the beam direction to allow for directional measurements of the field. B) The bi-planar geometry of the coil system. Two square planes of side length 2L = 1.6 m are placed a distance 2a = 1.5 m apart. Each coil produces a homogeneous field or field gradient (to within ±5%) inside the region highlighted by the green cube. C) Subject seated between the bi-planar coils wearing a scanner-cast. The subject-specific design of these casts allows the OPMs to be held in place with a known position and orientation with respect to the head, even during significant head movements.

As a result of these limitations, there is considerable interest in developing scalp-mounted MEG systems, and one particularly promising technology is the Optically-Pumped Magnetometer (OPM). OPMs use optical pumping of a heated vapour of spin-polarised alkali atoms to provide a measure of the local magnetic field (see Fig. 1A) (Kastler, 1973). Such systems offer many advantages compared to SQUID-based systems, including the possibility of flexible sensor placement on the scalp, a significant increase in sensitivity due to a reduction in the brain-to-sensor separation and potentially lower purchase and operating costs. The small and lightweight nature of OPMs also offers the potential for fabricating a “wearable” scalp-mounted MEG system that would allow recordings to be made while the subject makes large, natural head movements. Simulation studies (Boto et al., 2016; Iivanainen et al., 2017) have demonstrated the gains in performance which could be achieved using OPM-based MEG systems. Further, MEG measurements using a small number of OPMs have been experimentally realised and evoked responses following auditory or somatosensory stimulation have successfully been detected (Borna et al., 2017; Boto et al., 2017; Johnson et al., 2010, 2013; Sander et al., 2012; Xia et al., 2006). Additionally OPMs have been used to detect changes in alpha (8–13 Hz) and beta (13–30 Hz) oscillations (Boto et al., 2017; Kamada et al., 2015; Sander et al., 2012).

These experimental realisations of OPM-MEG have however involved recording neuromagnetic fields from restrained subjects, whose heads are fixed in position with respect to the sensors and surroundings. This limitation arises because the ambient magnetic field inside the OPM must be less than a few nT in magnitude if it is to operate with the sensitivity required for MEG, while the residual Earth's magnetic field inside a MSR used for conventional MEG is typically a few tens of nT. To avoid this problem, some OPM sensors contain a set of “on-sensor” coils which generate magnetic fields along three orthogonal directions within the vapour cell (Shah et al., 2018). These three on-sensor coils can be used to reduce the field within the cell from tens of nT to less than 1 nT, but such local coils produce fields which show a significant fractional variation in amplitude and orientation over the cell. Consequently, application of large cancellation fields reduces the sensor's sensitivity by making the field inhomogeneous over the heated vapour of spin-polarised atoms. More importantly, since the on-sensor coils compensate the ambient field for a specific location and orientation of the sensor, any translation or rotation of the sensor which produces a change of the vector field components that is greater than the nT dynamic range will result in saturation of the sensor output, rendering the data unusable (until the OPM returns to its original position).

Recently, we have shown that these problems can be avoided by reducing the remnant field inside the MSR using a larger set of fixed coils that are positioned around the entire OPM sensor array (Boto et al., 2018). In this approach, coils are mounted on two planes positioned on either side of the subject to form a bi-planar system, as shown in Fig. 1B. Unlike the tri-axial Helmholtz coil systems (Abbott, 2015) which are commonly used for field cancellation, this forms an open scanning environment, hence allowing easy access to the scanning area for the subjects and scanner operators. Our previous work showed that the integration of this coil system with a head-mounted OPM array allowed MEG data to be recorded whilst a subject made natural head movements, including head nodding, stretching and drinking tea (Boto et al., 2018). In the present paper, we describe an enhanced field-nulling coil system, incorporating six bi-planar coils and a 4-sensor reference array, and provide a full experimental demonstration of the system's performance. We begin by providing a detailed description of the design and construction of the bi-planar coils, using methods adapted from Magnetic Resonance Imaging (MRI). Specifically, mathematical expressions previously used for designing planar gradient coils (Yoda, 1990) were incorporated into a harmonic minimisation approach (Carlson et al., 1992; Turner, 1993). Following this, the efficacy of the resulting bi-planar coil set is characterised by mapping the residual static magnetic field vector inside a central region of the MSR, with and without the field nulling. We then demonstrate the extensive range of subject head motions that can be tolerated whilst maintaining operation of the OPMs, and show that residual magnetic artefacts in the resulting data can be markedly reduced by linear regression of head motion parameters that are measured using an infra-red camera system. Finally, we provide a unique neuroscientific demonstration of our system which involved instructing a subject to make head movements in order to shift the presentation of a visual stimulus across their visual field. By exploiting this novel means to capture the retinotopic organisation of the human visual cortex, we show that high fidelity, high spatial resolution MEG data can be measured even in the presence of large subject movements.

## 2 Methods

### 2.1 Theory of bi-planar coil design

Bi-planar field gradient coils have previously been employed in MRI to generate field gradients in a single component of the magnetic field vector (Haiying, 1998; Martens et al., 1991; Yoda, 1990). Here, the associated design methods were adapted to produce coils which compensate all three Cartesian components of the uniform background static field inside the MSR, as well as their spatial gradients. Initially it appears this would require 12 distinct coils (3 uniform field coils and 9 gradient coils). However, since both the divergence and curl of the magnetic field vanish in a current-free region there are only five independent field gradients. Therefore, 8 coils are needed to compensate for the three field components and their gradients. Based on analysis of the measured field variation in our MSR we have chosen to construct 6 coils (3 uniform field coils and 3 gradient coils) which together can produce an adequate reduction of the remnant field over a central region of the room.

To generate expressions that allow the design of biplanar coils, we consider the magnetic vector potential A(r) at position $r\left(x,y,z\right)$ due to current distribution J(r'), which is given by:

(1) $$A\left(r\right)=\frac{\mu_{0}}{4\pi }\int \frac{J\left(r^{'}\right)}{\left|r-\mathrm{r'}\right|}d^{3}\mathrm{r'}.$$

If the current is confined to the x-y plane at z=a, we can define the surface current density J in terms of a two-dimensional stream function S(x,y) such that $\nabla \text{S}\times \hat{z}=J$ (since ∇.J=0). Then, performing the Green's function expansion of ${\left|r-\mathrm{r'}\right|}^{-1}$ and re-writing the current density in terms of its two-dimensional Fourier transform, Eq. [1] can be re-formulated as:

(2) $$A\left(r\right)=\frac{i\mu_{0}}{2}\int_{-\infty }^{\infty }dk_{x}\int_{-\infty }^{\infty }dk_{y}e^{ik_{x}x}\;e^{ik_{y}y\;}\frac{e^{-k_{r}\left(z_{>}-z_{<}\right)}\;}{k_{r}}\left[k_{y}\hat{x}-k_{x}\hat{y}\right]\;\tilde{S}\left(k_{x},k_{y}\right),$$

where $\;k_{r}={\left(k_{x}^{2}+k_{y}^{2}\right)}^{1/2}$, $z_{>,<}$ is the greater or lesser of z or a and $\tilde{S}\left(k_{x},k_{y}\right)$ is the two-dimensional Fourier transform of the stream function. A bi-planar coil includes an equal or opposite current distribution confined to the plane at z=-a, so that $S_{z'=a}=\pm S_{z'=-a}$. Using Eq. [2] the magnetic field (B=∇×A) in the region between the planes, -a<z<a can be found by adding the contributions from the current distributions on the two planes:

(3) $$\tilde{B}\left(k_{x},k_{y},z\right)=\mu_{0}\left\{\left[ik_{x}\hat{x}+ik_{y}\hat{y}\right]\begin{array}{c} sinh\\ cosh \end{array}\left(k_{r}z\right)-k_{r}\hat{z}\begin{array}{c} cosh\\ sinh \end{array}\left(k_{r}z\right)\right\}\tilde{S}\left(k_{x},k_{y}\right)e^{-k_{r}a}.$$

here $\tilde{B}\left(k_{x},k_{y},z\right)$ denotes the two-dimensional Fourier transform of the magnetic field over the x−y plane at position z and the upper/lower sinh or cosh terms refer to the cases where the stream function has the same/opposite sign on each plane. The real-space field variation can be calculated from Eq. [3] via inverse Fourier transformation of $\;\tilde{B}\left(k_{x},k_{y},z\right)$.

To design a coil to produce a particular field variation, the stream function can be parameterised (Carlson et al., 1992), and then the parameter values which yield optimal performance based on a pre-defined functional can be identified. For the bi-planar coils considered here, the stream function is parameterised as a two-dimensional Fourier series which is confined to the region |x|,|y|<L (z=±a) on the two coil planes, so that:

(4) $$S\left(x,y\right)=\sum_{n=1}^{N}\left[\alpha_{n}\;\cos\left(\frac{\pi }{2}\left(2n-1\right)\frac{x}{L}\right)+\beta_{n}\;\sin\left(\frac{\pi nx}{L}\right)\right]\times \sum_{m=1}^{M}\left[\gamma_{m}\;\cos\left(\frac{\pi }{2}\left(2m-1\right)\frac{y}{L}\right)+\delta_{m}\;\sin\left(\frac{\pi my}{L}\right)\right],$$

where the coefficients $\alpha_{n}$, $\beta_{n}$, $\gamma_{m}$ and $\delta_{m}$ are used to weight the different harmonics in the series. Since the patterns of field variation that we aim to generate have a high degree of symmetry, only a sub-set of the harmonic combinations that arise from Eq. [4] are needed when designing each coil. For example, in the case of a $B_{x}$-coil, the stream function is required to be anti-symmetric in x, symmetric in y and anti-symmetric in z. These constraints allow the stream function to be written as

(5) $$S_{\left(B_{x}\right)}=\sum_{n=1}^{N}\sum_{m=1}^{M}\left[\lambda_{nm}\;\sin\left(\frac{\pi nx}{L}\right)\cos\left(\frac{\pi }{2}\left(2m-1\right)\frac{y}{L}\right)\right]\;$$

which defines the x and y symmetry, with the z symmetry defined by setting $S_{z=a}=-S_{z=-a}$. These stream function equations are written for ease of notation in the form $S=\sum_{j=1}^{N\times M}\lambda_{j}S_{j}$ with j=(n−1)N+m. Continuing with the case of the $B_{x}$-coil, the contribution to the field in the x-direction, $b_{xj}\left(r_{i}\right)$, from the $j^{th}$ component of the stream function can be expressed using Eq. [3] as

(6) $$\;b_{xj}\left(r_{i}\right)=2DFT{\left(i\mu_{0}k_{x}{\tilde{S}}_{j}e^{-k_{r}a}\;\cosh\left(k_{r}z_{i}\right)\right)|}_{x_{i},y_{i}},$$

where 2DFT indicates two-dimensional Fourier transformation.

Defining ${\tilde{S}}_{j}$ in terms of the reduced variables $x^{'}=x/L$, $y^{'}=y/L$ ($k_{x}^{'}=k_{x}L$ $k_{y}^{'}=k_{y}L$) allows its expression as

(7) S˜j(kx',ky')∝[sinc((n−12)π−ky')+sinc((n−12)π+ky')]×[sinc(mπ−kx')−sinc(mπ+kx')].

This can be substituted into Eq. [6] to find the field at position $r_{i}$ due to each component of the stream function. Similar calculations can be performed by imposing the symmetry conditions needed for the other coils (see Table 1).

**Table 1:** Summary of stream function symmetries required to produce a given magnetic field or field gradient. Symmetric (S) or Anti-Symmetric (A/S) terms can be extracted from Eq. [4] based on the x and y symmetry. Appropriate choice of sinh or cosh terms in Eq. [3] can be made using the z symmetry.

Coil	x symmetry	y symmetry	z symmetry
$B_{x}$	A/S	S	A/S
$B_{y}$	S	A/S	A/S
$B_{z}$	S	S	S
$dB_{x}/\mathrm{dz}$	A/S	S	S
$dB_{y}/\mathrm{dz}$	S	A/S	S
$dB_{z}/\mathrm{dz}$	S	S	A/S

Coil designs are produced by identifying the values of the λ-coefficients which minimise the functional (Carlson et al., 1992),

(8) $$F=\sum_{i=1}^{I}{\left|B_{x}\left(r_{i}\right)-b_{x}\left(r_{i}\right)\right|}^{2}+\omega P\;.$$

Here, $B_{x}\left(r_{i}\right)$ is the desired field at position $r_{i}$ and $b_{x}\left(r_{i}\right)=\sum_{j}\lambda_{j}b_{xj}\left(r_{i}\right)$ is the calculated field at $r_{i}$. The set of position vectors $r_{i=1\;\mathrm{to}\;I}$ define the target points within the volume at which a homogeneous field or field gradient is required. P is a tuneable power dissipation term which can be upweighted by increasing the weighting coefficient ω to reduce the complexity of the designed coils (Appendix A).

Here the functional is minimised by choosing the weights $\lambda_{j}$ which satisfy

(9) $$\frac{dF}{d\lambda_{j}}=0=-\sum_{i=1}^{I}B_{x}\left(r_{i}\right)b_{xj}\left(r_{i}\right)+\;\sum_{m}\lambda_{m}\left(\sum_{i=1}^{I}b_{xj}\left(r_{i}\right)b_{xm}\left(r_{i}\right)+\omega \text{Ω}\int_{-L}^{L}dk_{x}\int_{-L}^{L}dk_{y}\;k_{r}^{2}\;{\tilde{S}}_{j}{\tilde{S}}_{m}\right).$$

The set of derivatives can be cast as a set of linear simultaneous equations in matrix form, $\underline{\alpha }=\underline{\underline{\beta }}\underline{\lambda }$ with

(10) $$\alpha_{j}=\sum_{i=1}^{I}B_{x}\left(r_{i}\right)b_{xj}\left(r_{i}\right)$$

and

(11) $$\beta_{jm}=\sum_{i=1}^{I}b_{xj}\left(r_{i}\right)b_{xm}\left(r_{i}\right)+\omega \text{Ω}\int_{-L}^{L}dk_{x}\int_{-L}^{L}dk_{y}\;k_{r}^{2}\;{\tilde{S}}_{j}{\tilde{S}}_{m},$$

whose solution is found here by identifying the pseudo-inverse matrix. The wire paths of the coils are then extracted as contours of the optimised stream function.

### 2.2 Coil design and construction

Programmes were written in MATLAB (The MathWorks Inc.) to design coils based on the theory outlined in Section 2.1. Six coils were designed and constructed to allow nulling of the spatially-uniform field components, $B_{x}$, $B_{y}$ and $B_{z}$ and the three dominant, gradients of the field $dB_{x}/dz$ ($=dB_{z}/dx$), $dB_{y}/dz$($=dB_{z}/dy$) and $dB_{z}/dz$ ($=-2dB_{x}/dx=-2dB_{y}/dy$). Coils to generate the other gradients $dB_{x}/dy$ ($=dB_{y}/dx$) and $dB_{x}/dx$ ($=-dB_{y}/dy-dB_{z}/dz$) are described in Appendix B.

The dimensions of the coils were determined by the size and layout of the MSR which also contains a 275-channel (CTF, Coquitlam, BC, Canada) SQUID-based MEG system. These factors limited the final dimensions of the coils to a = 0.75 m and L = 0.8 m as shown in Fig. 1B. The coils were designed to produce homogeneous fields or gradients over a central volume of 40 × 40 × 40 cm^3^. The $B_{x}$, $B_{y}$, $dB_{x}/dz$ and $dB_{y}/dz$ coils were designed using 16 harmonics with N = M = 4 and the field was evaluated over I = 320 target points. The $B_{z}$ and $dB_{z}/dz$ coils were designed with 9 harmonics with N = M = 3 and the field was evaluated over I = 75 target points.

To allow construction of the coils from continuous wires, wire paths were formed with links inserted between the contours of the optimised stream function. Coils were mounted on two sheets of MDF measuring 1.8 × 1.8 m^2^ which were each attached to a support structure such that the centre of the coil set was raised by 1.1 m from the floor level of the MSR. This meant that with a seated subject the head-mounted OPMs would be located in the volume within which the coils generate uniform fields or field gradients. Coil designs were printed on paper sheets which were attached to the wooden boards using wallpaper paste. Enamelled copper wire of diameter 0.56 mm was laid on each printed path and fixed in place using masking tape. Additional coils were added in layers by repeating this procedure.

The two coils in each bi-planar coil pair were connected in series to a low-noise, 4 V, coil driver, which was controlled using a LabVIEW (National Instruments (NI) Corporation, Austin, TX) programme interfaced to a NI-9264 DAC voltage output module. An appropriately-sized resistor was added in series in each coil circuit so that a field of around 40 nT or field gradient of around 25 nT/m could be produced using the maximum voltage output of the coil driver (±4 V).

### 2.3 OPM sensors

Field measurements were made using commercially-available OPMs (QuSpin, Louisville, CO) which have a sensitivity of <15 fT/√Hz in the 1–100 Hz band, a dynamic range of ±1.5 nT and a bandwidth of approximately 1–130 Hz (Boto et al., 2018; Shah et al., 2018). The QuSpin sensor operates by shining circularly polarised, 795-nm-wavelength laser light onto a small cell containing a vapour of rubidium-87 (^87^Rb) atoms as shown in Fig. 1A. A photo-detector monitors the intensity of laser light transmitted through the cell. At zero magnetic field, the cell is relatively transparent and the photo-detector signal is a maximum. Under small applied fields the atoms undergo Larmor precession decreasing the transparency of the cell to the laser light. The photodetector output consequently shows a zero-field resonance with Lorentzian line shape (Dupont-Roc et al., 1969). Each QuSpin OPM contains a set of three coils which generate three orthogonal fields. These coils can be used to zero the static field components within the vapour cell up to a maximum value of ∼50 nT. We note that ‘field-zeroing’ refers to the on-sensor coils zeroing the field over the vapour cell on each OPM, whereas ‘field nulling’ refers to the bi-planar coils nulling the remnant field inside the MSR over the subject and OPM array. Sinusoidally-modulated magnetic fields of 1 kHz frequency are also applied perpendicular to the laser beam using the on-sensor coils. The phase of modulation of the transmitted light, which can be accurately monitored using a lock-in process, is sensitive to the magnitude of the field component along the modulation axis. Using this process, the amplitude of the two field components perpendicular to the laser can be simultaneously measured by applying oscillating currents to two coils in quadrature.

In addition to the standard measurement mode, the OPM sensors can also be operated in a “field-zeroing” mode. Here, the zero-field resonance is identified via lock-in detection of the sensor's response to an oscillating field applied perpendicular to the optical beam using one of the on-sensor coils. The strength of the zero-field resonance is automatically maximised by adjusting the DC currents in the three on-sensor coils (Shah and Hughes, 2015). Since the resonance is maximised when the field components perpendicular to the beam are zeroed, the magnitudes of the ambient field components oriented along two orthogonal directions perpendicular to the beam can be determined from the coil currents that maximise the signal, and the known field per unit current generated by the two relevant on-sensor coils. We used this field-zeroing procedure when mapping the ambient fields in the MSR.

Each sensor is contained within a 1.3 × 1.9 × 11 cm^3^ package with the sensitive volume located ∼6 mm from the outer surface (Fig. 1A). Subject-specific, 3D-printed scanner-casts as shown in Fig. 1C were used for the OPM-MEG measurements reported here (Boto et al., 2017). These casts contain slots which fix the positions and orientations of an array of the OPM sensors with respect to the head. The 40 × 40 × 40 cm^3^ volume of homogeneous field produced by the large bi-planar coils comfortably spans the OPM array when mounted in a scanner-cast (Boto et al., 2018).

Our previous work experimentally verified that cross-talk from currents applied to the on-sensor coils between OPMs at the sensor separations afforded by the scanner-casts is no more than 3%, which is deemed small enough to be ignored (Boto et al., 2018). As OPM arrays become more dense, the effects of cross-talk will become a growing problem and will require correction methods to be devised.

### 2.4 Automated field nulling and interference rejection

**Fig. 2:**
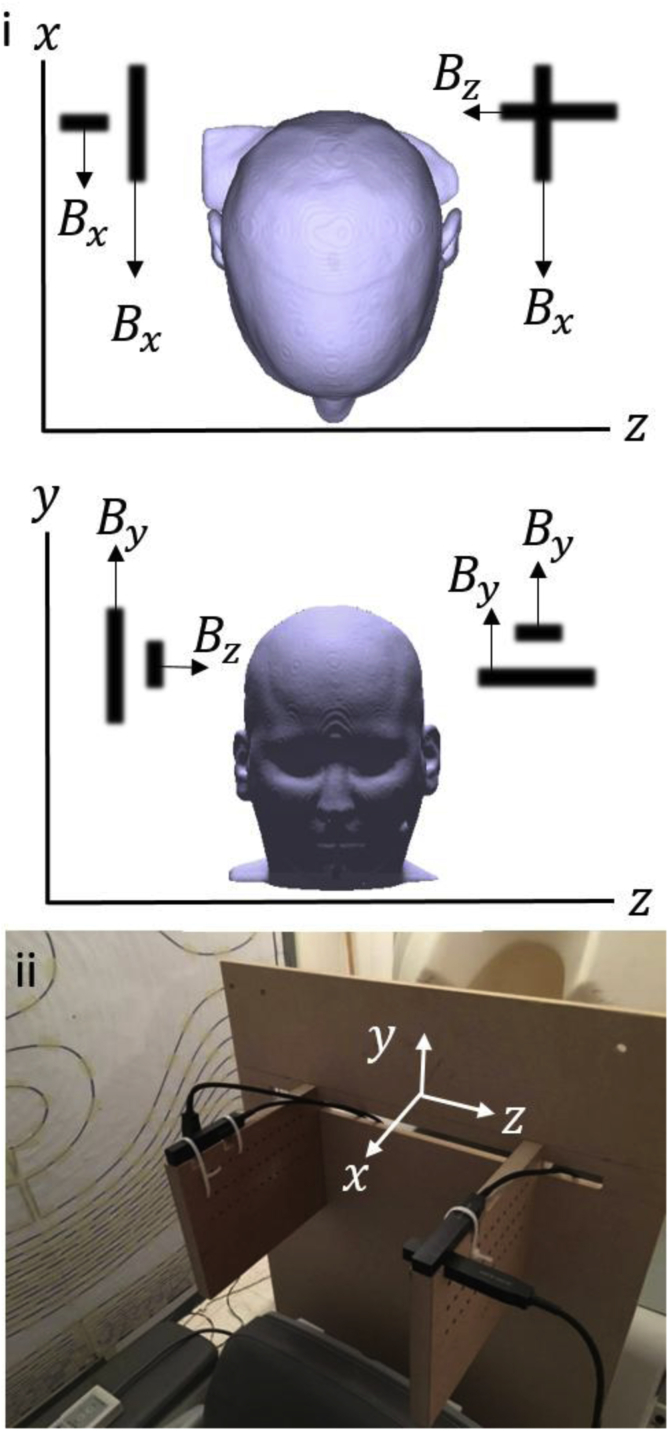
i) The positions and orientations of the four OPMs used for reference measurements. The OPMs are positioned such that a measurement of each field component can be made at two different z−positions to provide a measure of the field gradients. ii) The reference array as set up for an experiment.

In addition to the OPMs mounted within the scanner-cast, four OPMs were used to form a reference array which measured the ambient field within the MSR. Since the QuSpin OPMs have two sensitive axes of measurement, four OPMs could be used to measure the three field components $B_{x}$, $B_{y}$ and $B_{z}$, at two positions spatially-separated in the *z*-direction by ∼30 cm, as shown in Fig. 2. The output of these sensors provides information about the magnitude and spatial variation of the field components which was used in the field nulling process. A LabVIEW-based controller was developed to interface with the reference array and coil drivers. The static fields experienced by each sensor were measured by operating them in the field-zeroing mode (Shah and Hughes, 2015) and combined to form estimates of the magnitudes of $B_{x}$, $B_{y}$ and $B_{z}$ and of $dB_{x}/dz$, $dB_{y}/dz$ and $dB_{z}/dz$. These values were passed to Proportional Integral Derivative (PID) control loops which controlled the six coil current drivers.

Through operation of the PIDs, the field and gradient measurements were driven towards zero (in practice, reaching values of around 10 pT and 50 pT/m, respectively). Once nulling was completed the PIDs were switched off, and stable currents that were optimised for field nulling were applied to the coils. The nulling sensors could then be set up in their normal measurement mode and used as reference sensors: they were placed close enough to the head to monitor variations in the background interference during the course of an experiment, but far enough away to be relatively insensitive to magnetic field from the brain. This meant that a synthetic gradiometer could be formed, by applying linear regression to the brain data using the outputs of the reference sensors as predictors (Boto et al., 2017).

### 2.5 Field mapping

The reductions in the fields, and field gradients afforded by the coil were quantified by mapping the field in a single plane before and after field nulling, using the automated procedure described above. The field was mapped over a central 20 × 20 cm^2^ region in the *x*-*z* plane which was centred between the reference sensors (which were separated by 30 cm in the *z*-direction). By recording the output of the OPM on-sensor coils operating in the field-zeroing mode, measurements of static background field were taken with a single OPM sensor. The sensor was placed at two different orientations at each position on a 5-cm grid to obtain a representation of the spatial variation of the three Cartesian components of the static magnetic field. The field was then nulled using the bi-planar coils and the process repeated. The data were interpolated onto a 1-cm resolution grid and displayed as field maps to show the reduction in field strength and spatial variation of the field over the 20 × 20 cm^2^ plane, afforded by the field nulling coils.

### 2.6 Field stability

We devised a simple experiment to demonstrate the stability of the field nulling over time. An array of 7 OPMs was placed inside an empty scanner-cast positioned between the reference sensors. An initial measurement of the static background field was taken with the nulling system switched off. The field nulling was then performed as usual, and the currents applied to the bi-planar coils were set and then held constant. The magnitude of the static background field was measured every 5 min over a 30-min period using the on-sensor coils of the 7 OPMs. The OPMs were then switched off and the door to the MSR opened (none of the experimental equipment was displaced) for 30 min, while the currents in the bi-planar coils were still held constant. The sensors were then rebooted and the door to the MSR closed, and the static field was measured once every 5 min over an additional 30-min period.

The power spectral density of the OPM signals were also investigated with the field nulling system on and off. A single OPM in the empty scanner-cast was chosen and recordings were completed over 60-s periods. For these measurements, only the radial component of the magnetic field was recorded with a sampling frequency of 1200 Hz. Environmental interference is prominent in these recordings as no interference-reduction methods have been applied to these magnetometer recordings; any additional interference produced by the coils and their associated electronics is therefore easy to identify.

### 2.7 Demonstrating the allowed range of motions

**Fig. 3:**
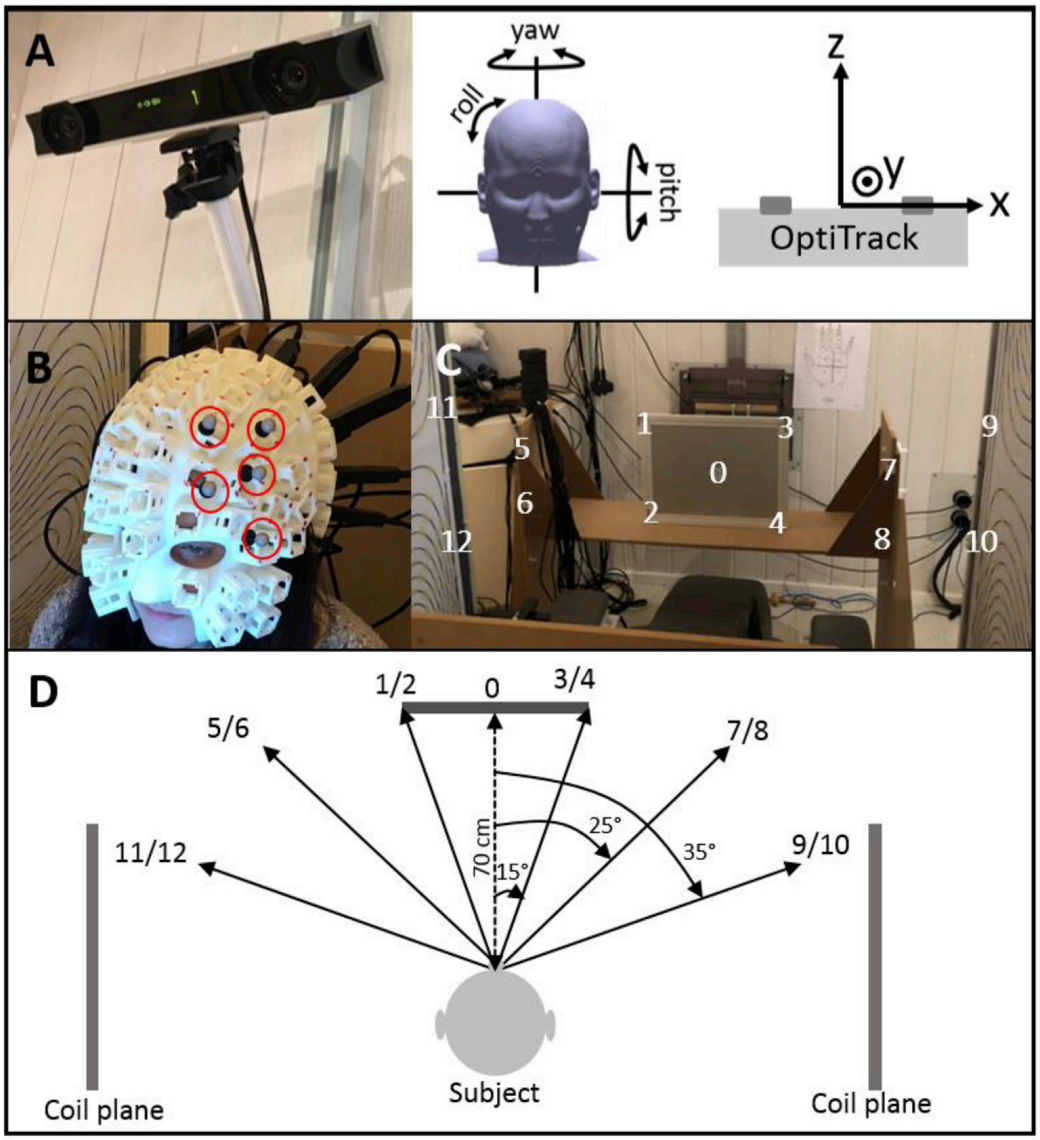
A) The OptiTrack V120:Duo optical tracking camera is used to measure subject movement. The translations (*x*, *y*, *z*) and rotations (pitch, yaw, roll) are measured in the camera's frame of reference. B) The system uses infra-red LEDs to illuminate a series of 5 highly reflective markers (circled in red) positioned on the surface of the scanner-cast. These markers are combined to form a single rigid-body which is then tracked during an experiment. C) A series of 13 numbered marks were positioned within the field of view of the subject. The subject was instructed to shift their gaze to these marks. D) Approximate angular movements of the head required to shift the gaze to each mark.

To monitor the movement of the subject's head during experiments, motion data were captured using an OptiTrack V120:Duo camera system (NaturalPoint Inc., Corvallis) which provides sub-millimetre and sub-1-degree precision optical tracking of a rigid body with six degrees of freedom: translations (*x*, *y*, *z*) and rotations (pitch, yaw, roll) as shown in Fig. 3A. The system comprises two cameras, each of which has an associated array of 15 infrared LEDs that are used to illuminate multiple, highly reflective markers, which were attached to the scanner-cast as shown in Fig. 3B. The camera system initially identifies the individual marker positions and uses their combined co-ordinates to define a rigid body; rotation and translation of the rigid body can then be monitored at the camera's 120 Hz frame rate. In these experiments, the rigid body was formed from 5 markers.

We devised a simple experiment to demonstrate the wide range of head movements that a subject could make when the field nulling system was in operation, without causing any OPM sensor in a head-mounted array to go outside its ±1.5 nT operating range. Thirteen visual marks (numbered from 0 to 12) were fixed either to a bench, placed 70 cm in front of the subject, or to the edges of the two coil planes, as shown in Fig. 3C–D. Marks 0–4 were sited close to the centre of the subject's field of view when they were seated and looking directly forwards. Marks 5–8 were positioned so that the subject would have to rotate their head approximately ±25° from the centre point to bring them to the centre of their field of view. Marks 9–12 required larger head rotations of around ±35° from the centre point. The movements required to view marks 9–12 were at the limit of what was deemed comfortable for the subject to achieve without rotation of the body. To enable a controlled assessment of the effect of these motions on OPM recordings, the subject was first asked to focus on mark 0 (central) and then after 10 s the subject was instructed to switch their gaze to mark 1. After a further 10 s the subject was instructed to look at mark 2 etc. After 4 markers had been viewed the subject was asked to return to viewing mark 0 before beginning the viewing of the next 4 marks. The full experiment lasted 160 s.

Magnetic field data were simultaneously recorded from an array of 18 OPMs which were mounted in the scanner-cast to provide good coverage of the visual cortex. Note that we chose the visual cortex particularly because the yaw of the head required to fixate on the markers would generate an exaggerated movement of the sensor locations at the back of the head, hence providing the most challenging setting in terms of OPM movement. Recordings of the sensor outputs were made using a set of 3 NI-9205 Data Acquisition cards sampling at 1200 Hz. In these experiments, only the fields radial to the head surface were measured. At the start of the experiment, currents through the bi-planar coils were optimised to null the ambient fields as discussed previously. The residual fields at each sensor were then zeroed using the on-sensor coils. Following this, the MEG recording was initiated and the subject was instructed to start making the movements.

As the magnetic fields reported by the OPMs are affected by interference from fluctuating background fields, as well as field changes due to subject motion in the remnant Earth's field, multiple linear regression analyses were used to attenuate these confounds (Boto et al., 2017). A design matrix of predictors was formed using the 6 motion parameters (up-sampled through linear interpolation to an effective sampling rate of 1200 Hz), as well as the signals from the 4 reference magnetometers discussed previously. Regression was performed over the full duration of the experiment.

### 2.8 OPM-MEG demonstration: measurement of retinotopy: data acquisition

To test the fidelity of the MEG data captured with head movement following field nulling, a novel means to exploit the retinotopic organisation of the visual cortex was devised. Traditionally in retinotopic mapping experiments (e.g. Engel et al., 1997) the subject remains still with their gaze fixed centrally, while a stimulus is moved around the subject's field of view. Analysis then reveals visual cortex activation in different locations depending on the position of the stimulus in the visual field. In the simplest case, a stimulus presented in the right visual hemifield will evoke activity in left visual cortex, and vice-versa. Here, taking advantage of the head movement allowed by the field-nulling bi-planar coil set, we aimed to demonstrate this simple aspect of retinotopic mapping, by making measurements with the stimulus fixed in position while the subject physically moves their gaze by head rotation/translation.

Presentation (Neurobehavioral Systems Inc., Berkeley, CA) was used to generate a checkerboard pattern reversing at 4 Hz, which was expected to produce a driven, 8 Hz, electrophysiological response in the visual cortex. This pattern was projected onto a screen positioned 50 cm away from the subject's eyes. The checkerboard had dimensions of 10 × 10 cm^2^ and subtended a visual angle of 11° × 11°. Each check had dimensions of 2 × 2 cm^2^ and subtended a visual angle of 2.3° × 2.3°. Two crosses were placed on the top corners of the stimulus presentation screen as fixation points, so that when the subject fixed their gaze on these crosses the stimulus would be in either the lower right, or lower left, quadrant of their visual field. Magnetic field data were recorded from an array of 18 OPMs which were positioned to provide good coverage of the visual cortex and each sensor measured a single field component that was radial to the head surface.

The timing of the stimulus was such that a single trial lasted for 8 s. The reversing checkerboard was on for 3 s (16 cycles) followed by a 3 s rest period during which the screen showed only a central cross. In the final 2 s of each trial the words “switch gaze” were shown on the screen. The subject was instructed to either choose whether to switch their gaze to the opposite side of the screen, or to remain still during this period. There were 80 trials in total, and importantly, at the time of acquisition, in any one trial the experimenters did not know which of the two crosses the subject was fixated on (and hence they didn't know in which visual hemifield the stimulus was located). To address this issue, the subject's head location and orientation were recorded throughout the experiment using the OptiTrack camera. Prior to MEG recording, a calibration measurement was performed in which the subject was simply asked to focus on the two crosses in turn.

### 2.9 OPM-MEG demonstration: measurement of retinotopy: data analysis

The OPM-MEG data acquired during our retinotopy experiment were processed using a beamformer approach. Using information from the OptiTrack camera, the data were segmented into two sub-sets comprising trials where the stimulus was on the left, or on the right. Regression of the data with motion parameters and the signals from the reference magnetometers was performed on a trial-by-trial basis. Data were then frequency-filtered between 4 Hz and 12 Hz. The resulting two subsets of data were then averaged over trials. For left visual stimulus presentation, 3 covariance matrices were derived: $C_{\mathrm{la}}$ represented data covariance in the (0 s < t < 3 s) active time window (when the stimulus was on), $C_{\mathrm{lc}}$ represented data covariance in the (3 s < t < 6 s) control time window (when the stimulus was off, but before any movement) and $C_{l}=\frac{C_{\mathrm{la}}+C_{\mathrm{lc}}}{2}$ was simply the average of the two, representing the whole trial before gaze shifting. Three equivalent covariance matrices, $C_{\mathrm{ra}}$, $C_{\mathrm{rc}}$ and $C_{r}$, were constructed for data recorded during right-hemifield stimulation. $C_{l}$ and $C_{r}$ were both regularised using the Tikhonov method to produce $C_{l\;\mathrm{reg}}$ and $C_{r\;\mathrm{reg}}$, with the regularisation parameter set to 0.01 times the maximum eigenvalue of the unregularised matrix. Independent beamformer weights were constructed for each sub-set of data, such that for any one source space location and orientation, θ,

(12) $$w_{l\theta }^{T}=\frac{l_{\theta }^{T}C_{l\;reg}^{-1}}{l_{\theta }^{T}C_{l\;reg}^{-1}l_{\theta }}\;and\;w_{r\theta }^{T}=\frac{l_{\theta }^{T}C_{r\;reg}^{-1}}{l_{\theta }^{T}C_{r\;reg}^{-1}l_{\theta }}$$

here, $w_{l\theta }$ and $w_{r\theta }$ represent beamformer weights tuned to left and right stimuli respectively. Note that data averaging prior to covariance calculation ensures that the weights are tuned to the trial-averaged steady state response of interest (Brookes et al., 2010). Further, computing separate weights for each data subset maximises the spatial specificity of the resulting beamformer images (Barratt et al., 2018). Two separate pseudo-T-statistical beamformer images were then derived as

(13) $${Ŧ}_{\mathrm{l\theta}}=\frac{w_{l\theta }^{T}C_{\mathrm{la}}w_{l\theta }-w_{l\theta }^{T}C_{\mathrm{lc}}w_{\mathrm{l\theta}}}{2w_{l\theta }^{T}w_{l\theta }}\;and\;{Ŧ}_{\mathrm{r\theta}}=\frac{w_{r\theta }^{T}C_{\mathrm{ra}}w_{r\theta }-w_{r\theta }^{T}C_{\mathrm{rc}}w_{\mathrm{r\theta}}}{2w_{r\theta }^{T}w_{r\theta }}$$

Here, ${Ŧ}_{\mathrm{l\theta}}$ is an image of the spatial signature of evoked (8 Hz) brain activity when the stimulus was on the left, and ${Ŧ}_{\mathrm{r\theta}}$ represents equivalent 8 Hz activity with the stimulus on the right. Both images were computed at the vertices of a regular 2 mm grid spanning the whole source space (i.e. the brain). A spherically symmetric conductor was assumed. The magnetic field outside the sphere due to a current dipole inside was calculated using the analytical formula introduced by Sarvas (Sarvas, 1987). Since in this model a radial source produces no magnetic field, the source orientation for the beamformer was selected in the plane tangential to the radial direction to yield the highest beamformer output for each location probed.

Based on these pseudo-T-statistical images, two locations of interest were selected (1 and 2), and beamformer reconstructed signals were calculated for both, using the two data sub-sets. This resulted in 4 “virtual electrode” time courses: if $m_{r}\left(t\right)$ and $m_{l}\left(t\right)\;$ represent the trial averaged MEG data recorded when the stimulus was in the right and left visual fields, respectively; $q_{r1}\;(=w_{r1}^{T}m_{r}\left(t\right))$ represents the time course of electrical activity at location 1, during right hemifield stimulation and $q_{r2}\;(=w_{r2}^{T}m_{r}\left(t\right))$ represents activity at location 2, during right hemifield stimulation. Similarly, $q_{l1}\;(=w_{l1}^{T}m_{l}\left(t\right))$ represents the time course of electrical activity at location 1, during left hemifield stimulation and $q_{l2}\;(=w_{l2}^{T}m_{l}\left(t\right))$ represents activity at location 2, during left hemifield stimulation. All 4 of these time courses were Fourier transformed, and then tested for the expected peak at 8 Hz. We hypothesised that peak locations 1 and 2 would appear in the contralateral (right and left) hemispheres in response to left and right-sided visual stimulation, respectively. Further we hypothesised that the 8 Hz response would only be observed in contralateral, and not ipsilateral visual cortex.

## 3 Results

### 3.1 Coil designs

**Fig. 4:**
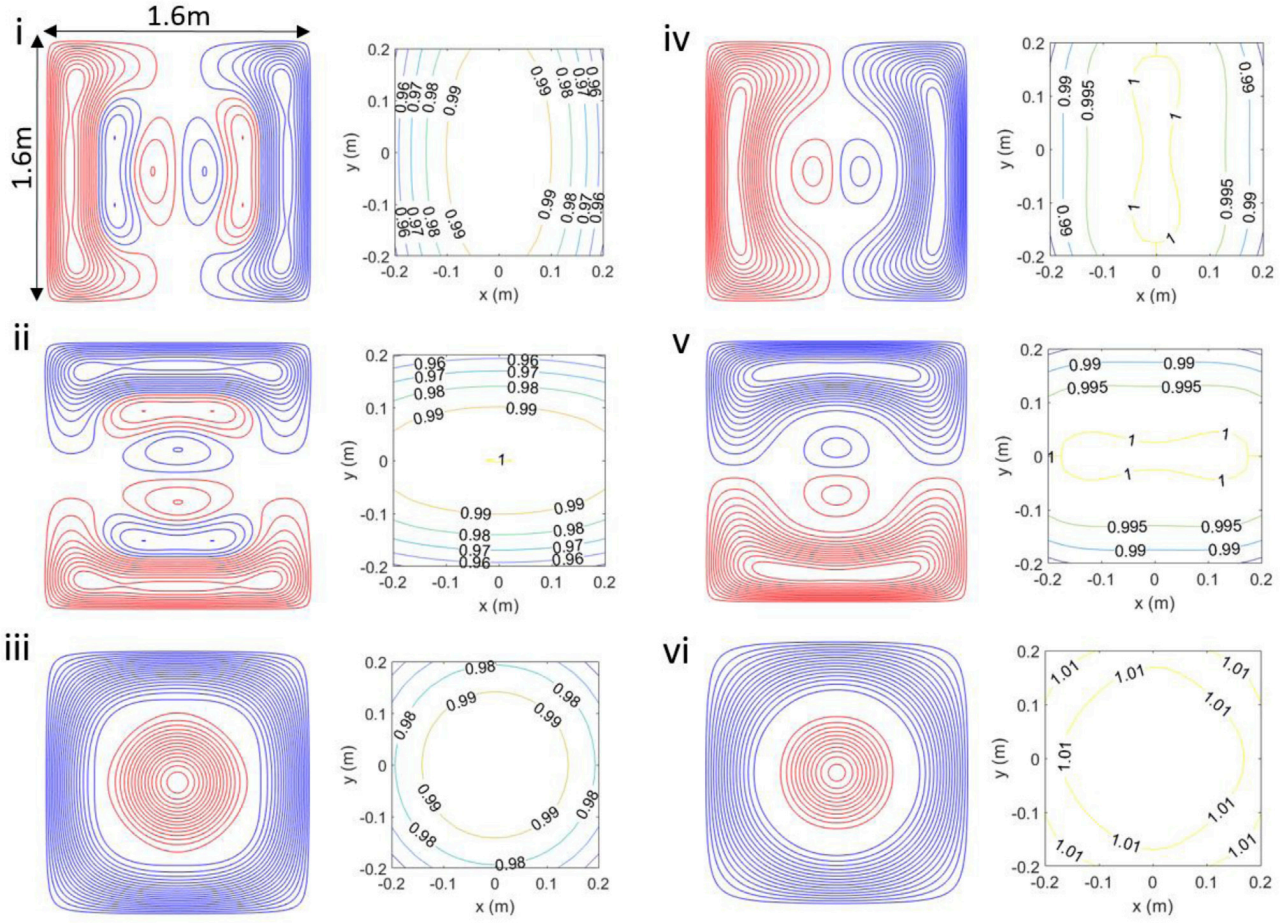
Wire paths and field or field gradient contours for the: i) $B_{x}$, ii) $B_{y}$, iii) $B_{z}$, iv) $dB_{x}/dz$, v) $dB_{y}/dz$, vi) $dB_{z}/dz$ coils. Red and blue denote wires with opposite senses of current flow. The contours of the field (i-ii) or field gradient (iv-vi) in the plane at z = 0 m, for |*x*|, |*y*| < 0.2 m (*z* = 0.02 m, for |*x*|, |*y*| < 0.2 m for the $dB_{z}/dz$ coil) are shown. The fields were normalised to the value at the centre of the two planes (or a value positioned off-centre where the field gradient is zero at the centre).

Fig. 4 shows the wire paths and contours of the spatial variation of the field or field gradient for each of the six coils ($B_{x}$, $B_{y},\;B_{z}$, $dB_{x}/dz$, $dB_{y}/dz$ and $dB_{z}/dz$) that were constructed. Red and blue colours denote opposite senses of current flow in the coil windings. The field variation produced by each coil was calculated by applying the elemental Biot-Savart expression to the digitised wire paths. The variation of the field or field gradient relative to the value at the centre of the coils (or at a value positioned slightly off centre for field gradient coils since the field is zero at the centre for these coils) was then evaluated and contoured as a measure of the homogeneity of the fields generated by the coils. Fig. 4 shows contours in the plane at z = 0 m; |*x*|, |*y*| < 0.2 m for the $B_{x}$, $B_{y},\;B_{z}$, $dB_{x}/dz$, and $dB_{y}/dz$ coils, and in the plane z = 0.02 m; |*x*|, |*y*| < 0.2 m for the $dB_{z}/dz$ coil. The deviation from the desired pattern of field variation was found to be less than 5% within a central region of 40 × 40 × 40 cm^3^ extent for all coils. Designs for the additional two coils ($dB_{x}/dx$ and $dB_{y}/dx$) are included in Appendix B.

Table 2 shows the calculated and measured coil parameters for the six coils that were constructed. Calculation of coil resistance values assumed that they were wound using 0.56-mm-diameter copper wire. The coil inductances were calculated from the stream functions using previously published expressions (Martens et al., 1991).

**Table 2:** Bi-planar coil characteristics: theoretical results for each coil are shown with measured values in brackets. The calculated values of resistance assume that 0.56 mm diameter copper wire was used in coil construction.

Coil	Length of wire (m)	Resistance of wire (Ω)	Coil Strength (nT/mA or nT/m/mA)	Inductance (μH)
$B_{x}$	170	11.9 (14.2)	1.21 (1.13 ± 0.1)	544 (619)
$B_{y}$	170	11.9 (13.3)	1.21 (1.06 ± 0.1)	544 (614)
$B_{z}$	186	13.0 (13.8)	7.29 (7.90 ± 0.3)	1520 (1290)
$dB_{x}/\mathrm{dz}$	195	13.7 (16.1)	6.75 (6.50 ± 0.2)	843 (985)
$dB_{y}/\mathrm{dz}$	195	13.7 (15.5)	6.75 (6.33 ± 0.6)	843 (984)
$dB_{z}/\mathrm{dz}$	168	11.8 (12.8)	14.4 (14.0 ± 1.0)	968 (1090)

### 3.2 Automated field nulling

**Fig. 5:**
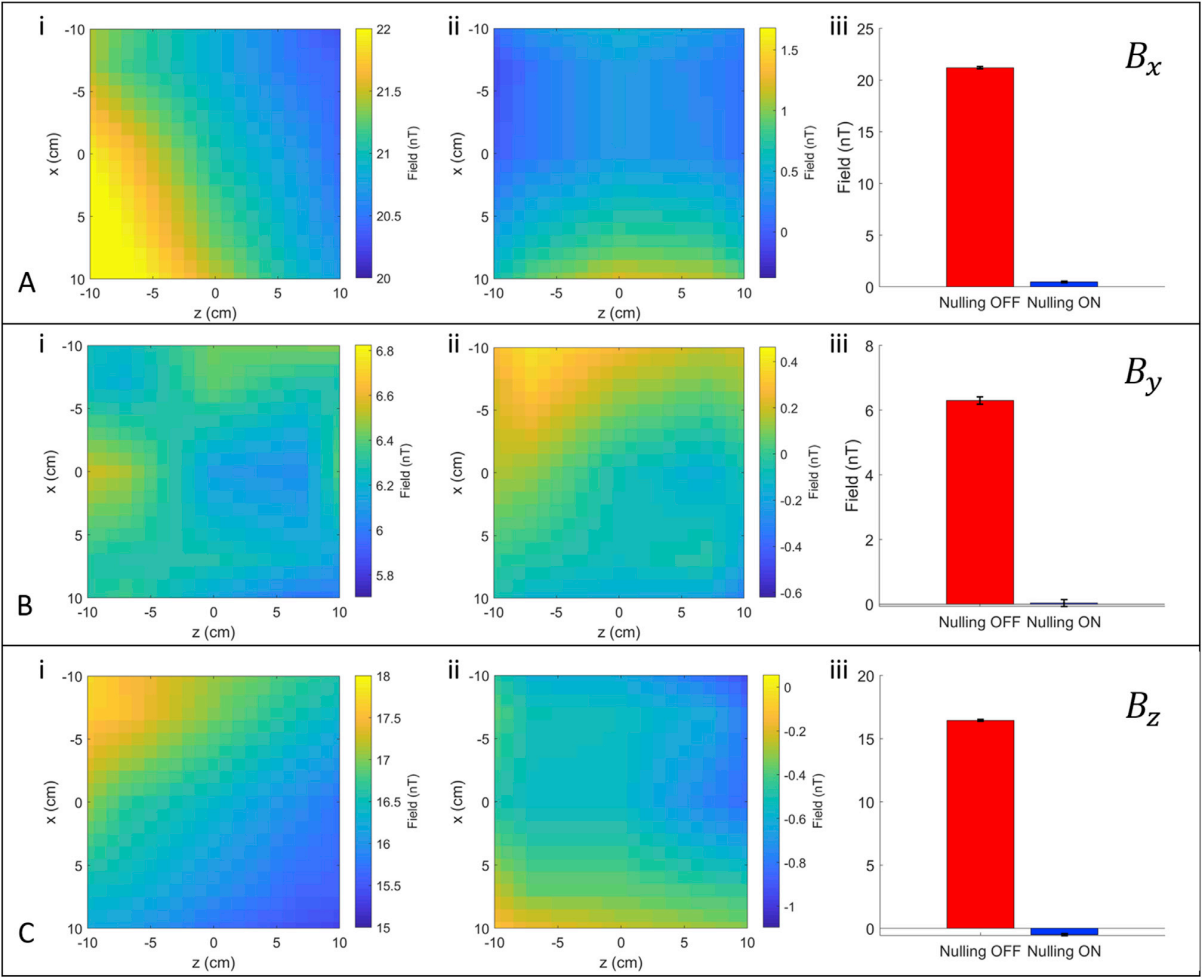
A) B) and C) show for $B_{x}$, $B_{y}$ and $B_{z}$ respectively i) a map of the field before nulling is applied ii) a map of the field after nulling is applied and iii) the average field magnitude with error-bars showing the standard deviation of measurement before (red) and after (blue) nulling is applied.

Fig. 5 shows field maps measured before (i) and after (ii) the automated nulling was applied and a bar plot of reduction in average field strength (iii), as measured using the field-zeroing procedure with a single OPM sequentially sampling field components at different positions on a reference grid with 5-cm grid spacing. The mean magnitude of the field vector $\left|B\right|=\sqrt{B_{x}^{2}+B_{y}^{2}+B_{z}^{2}}$ fell from 28.0 nT to 0.74 nT, corresponding to a reduction by a factor of 38 (averaged over the full 20 × 20 cm^2^ plane studied). In terms of spatial field variation, the root mean square deviation from the mean value fell from 0.6 nT to 0.16 nT, a reduction by a factor of ∼4.

### 3.3 Field stability

**Fig. 6:**
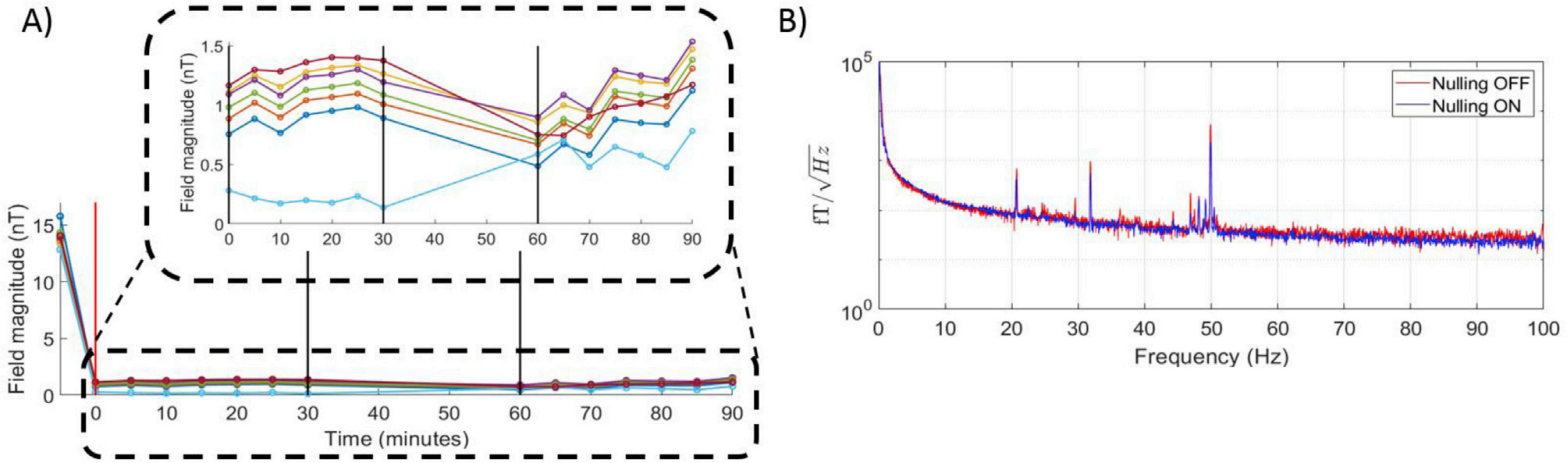
A) The static field magnitude as reported by the on-sensor coils for an array of 7 OPM sensors at 5-min intervals over a 30 min period. The red line shows when the field nulling was applied, the black lines show a period where the sensors were switched off, and the door to the MSR was left open. There followed an additional 30 min where the door was closed and further field measurements were made. The bi-planar coil currents were held constant throughout the entire 90-min experiment. B) The noise power spectra of a single OPM in this scanner-cast recorded with the field nulling OFF and ON.

The measured field magnitude is plotted as a function of time in Fig. 6A. Analysis of these data shows that the maximum change in field magnitude over the 90 min on any sensor was 0.50 nT, while the average (over all 7 sensors) of the standard deviation over time was 0.18 ± 0.03 nT. These values are both smaller than the ±1.5 nT dynamic range of the OPMs, showing that the field nulling achieved was sufficiently stable over the duration of our experiments.

The power spectral density of the OPMs with the field nulling on (red) and off (blue) are shown in Fig. 6B. There is little difference in the two measurements when the OPMs remain still.

### 3.4 Demonstrating the allowed range of motions

**Fig. 7:**
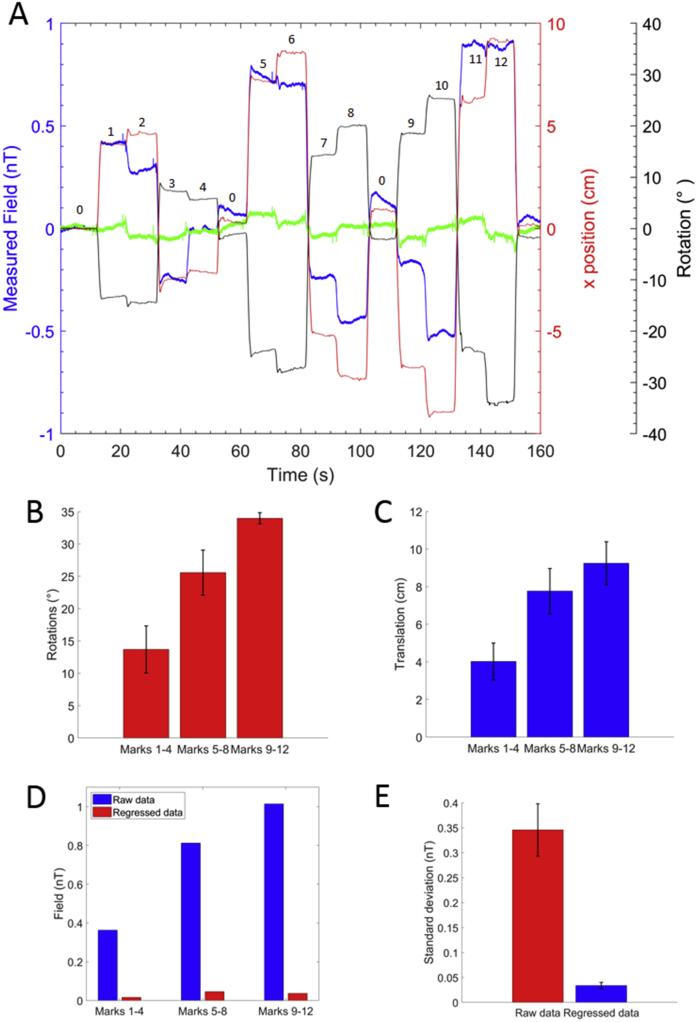
A) The output of a single OPM within an array of 18 worn by the subject is displayed in blue. The magnetic field data are then compared with the *x* translation (red) and yaw rotation (black) of the rigid body. The text labels identify the order in which the positions were viewed during the experiment. Even for the largest motions, which were at the limit of what was deemed comfortable for the subject, the sensors stayed within their ±1.5 nT dynamic range. The measured field following regression with motion parameters and the four reference magnetometers is shown in green. B) The magnitude ($\sqrt{pitch^{2}+roll^{2}+yaw^{2}}$) of the rotation required to move from mark 0 to each mark is averaged over the 3 subsets. C) The magnitude ($\sqrt{x^{2}+y^{2}+z^{2}}$) of the translation required to move from mark 0 to each mark is averaged over 3 subsets each containing 4 marks. D) Reduction in field following regression at each subset of marks shown for the sensor which detected the largest (positive) field over the whole experiment. E) Reduction in the standard deviation over time of raw sensor outputs during the entire 160 s of the experiment following regression. Results are averaged over all 18 sensors in the scanner-cast.

Fig. 7A shows the output of a single OPM (blue) as the subject moved their head. Measurements of the translation (in the *x*-direction) and rotation (yaw) of the head that were simultaneously recorded by the OptiTrack are also plotted in red and black, respectively, for comparison. The output of the OPM following linear regression with the 6 motion parameters and the output of the 4 reference sensors is shown in green. It is evident that the sensors remain within their operational range (±1.5 nT) even after the largest movements when field nulling is applied. Fig. 7B and C shows bar charts which describe the magnitude of the total rotations ($\sqrt{pitch^{2}+roll^{2}+yaw^{2}}$) and translations ($\sqrt{x^{2}+y^{2}+z^{2}}$) recorded by the OptiTrack for three subsets of marks (1–4, 5–8, 9–12). The associated root mean squared deviations from the mean for each case are also shown. Fig. 7D shows the reduction in size of the measured field following regression at each subset of marks for the sensor which detected the largest positive field over the whole experiment. Fig. 7E shows the reduction in standard deviation over time for the entire experiment following regression, averaged over all 18 sensors. The largest head motion was produced when the subject viewed marks 9–12, requiring a rotation of ±34.0 ± 0.9° and translation of ±9.2 ± 1.1 cm from the central position. This motion produced a maximum field artefact of approximate magnitude 1 nT, which was reduced following regression with the motion parameters to 0.037 nT.

### 3.5 OPM-MEG demonstration: measurement of retinotopic mapping

**Fig. 8:**
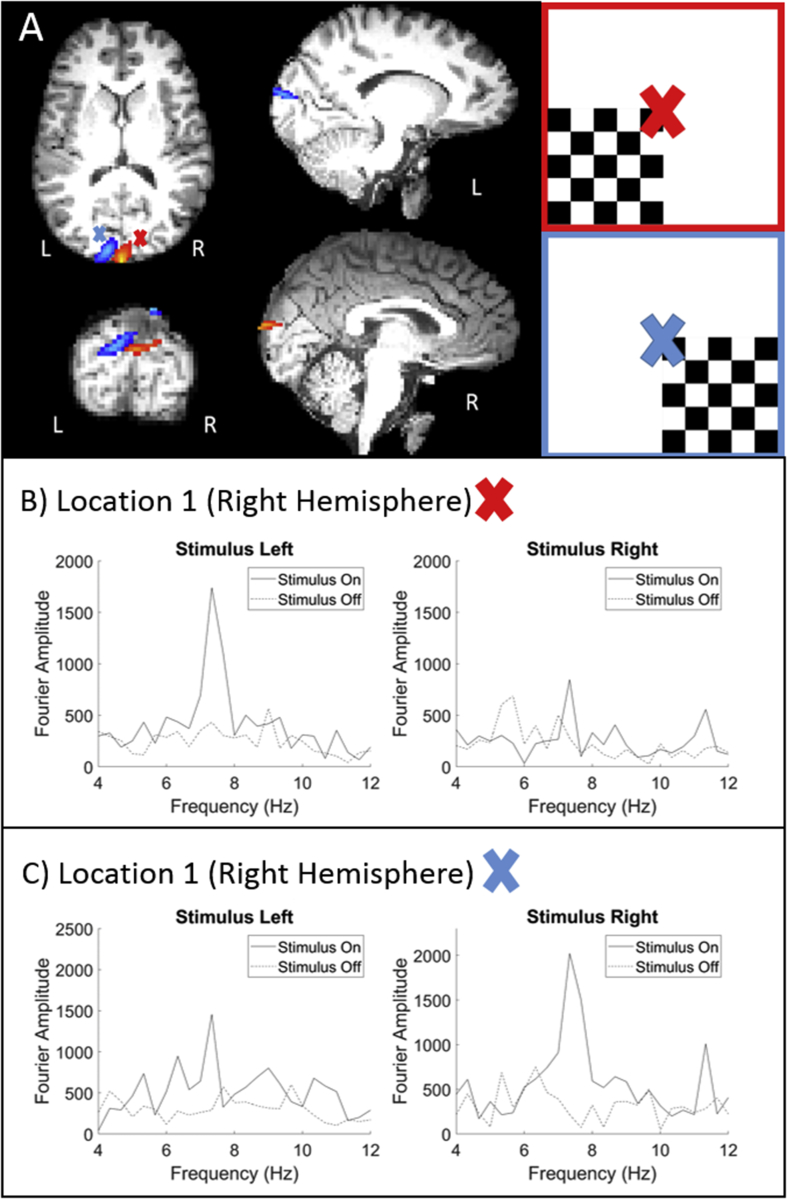
A) Pseudo T-stat images produced from the cases where the stimulus was in the left visual field (red cross, red overlay, subject view shown in red inset) or the right visual field (blue cross, blue overlay, subject view shown in blue inset). The images were thresholded between 1.5 and 1.8 and 1.1 and 1.6 for the red and blue images respectively. B) The Fourier transforms of virtual electrode time-courses compared during stimulation and rest. The electrode was positioned at the peak of the red overlay and compared for the cases where the stimulus was on the left and on the right. C) Comparing Fourier transforms of virtual electrodes positioned at the peak of the blue overlay and compared for the cases where the stimulus was on the left and on the right.

Fig. 8A shows the pseudo-T-statistical images where the red/blue overlays depict the beamformer image formed when the stimulus was on the left/right. Insets highlighted in red/blue show the subject's view. Evaluating the motion tracking data during the “switch gaze” period for the trials where the subject moved revealed the magnitude of translations and rotations to be 1.4 ± 0.04 cm and 5.9 ± 0.8°, respectively.

Fig. 8B (i) compares Fourier transforms of the virtual electrode time courses produced during stimulation and rest periods at location 1 when the stimulus was on the left ($q_{l1}$). Fig. 8B (ii) compares Fourier transforms of the virtual electrode time courses produced during stimulation and rest at location 1 when the stimulus was on the right ($q_{r1}$). Fig. 8C (i) compares Fourier transforms of the virtual electrode time courses produced during stimulation and rest periods at location 2 when the stimulus was on the left ($q_{l2}$). Fig. 8C (ii) compares Fourier transforms of the virtual electrode time courses produced during stimulation and rest at location 2 when the stimulus was on the right ($q_{r2}$).

As expected, the areas of largest 8 Hz response in the beamformer images for the two cases localise to opposite sides of the visual cortex. Inspecting the Fourier transforms there are clear 8 Hz responses during periods of stimulus presentation in the corresponding cortex when compared to rest. Looking in the opposing cortex during stimulus presentation reveals no response.

## 4 Discussion

### 4.1 Bi-planar coils

By adapting coil design methods that have previously been used for producing gradient coils for MRI, we have designed a set of six bi-planar coils that can be used to null the residual magnetic fields and the dominant magnetic field gradients over a 40× 40 × 40 cm^3^ volume within a MSR. Field nulling can be accomplished by using an automated procedure which relies on measurements of the three Cartesian components of the magnetic field made at two locations using four OPM sensors (each of which measures two orthogonal components of the magnetic field), as shown in Fig. 2. Using PID loops implemented in software, it takes around 20 s to establish the current levels in the six coils that produce the maximal reduction in the magnetic fields at the reference sensors. The maximum values of the total field magnitude $\left|B\right|=\sqrt{B_{x}^{2}+B_{y}^{2}+B_{z}^{2}}$ across the 20 × 20 cm^2^ central *x*-*z* plane before and after nulling are found to be 29 nT and 1.2 nT respectively.

Operating inside our MSR, which is formed from two layers of mu-metal sandwiching one layer of aluminium, the bi-planar coil set reduced the largest residual uniform field component, $B_{x}$, from 21.8 ± 0.2 nT to 0.47 ± 0.08 nT (reduction by a factor of 46) over the 20 × 20 cm^2^ central *x*-*z* plane (Fig. 6), and the largest gradient component, $dB_{x}/dz$ from 7.4 nT/m to 0.55 nT/m (reduction by a factor of 13). The $B_{y}$ component was reduced from 6.3 ± 0.1 nT to 0.03 ± 0.10 nT (reduction by a factor of 210), and the $dB_{y}/dz$ gradient was reduced from 2.8 nT/m to 0.50 nT/m (reduction by a factor of 6). The $B_{z}$ component was reduced from 16.4 ± 0.1 nT to −0.49 ± 0.08 nT (reduction by a factor of 33), and the $dB_{x}/dz$ gradient was reduced from 2.8 nT/m to 0.50 nT/m (reduction by a factor of 6).

The field nulling reported here was accomplished with currents of less than 30 mA running in the coils, since the coil efficiencies are in the range of 1–8 nT/mA and 6–14 nT/m/mA for the uniform field and gradient coils, respectively (see Table 2). As the coil resistances are of the order 10 Ω, the maximum voltages applied to the coils were around 0.3 V, and the maximum power dissipated in each coil was less than 10 mW. Although we did not drive time-varying currents through the coils in this work, simple analysis shows that it would be possible to generate rates of change of field (field gradient) that are greater than 7 nT/ms (25 nT/m/ms) with just 4 V driving voltage (based on a calculation of $\frac{dB}{dt}or\frac{dG}{dt}=\eta V/L\;$, where η is the coil efficiency). This would readily allow the bi-planar coil system to be used in future work to cancel time-varying fields from interference sources located outside the MSR, since these fields are generally much smaller in magnitude than the remnant Earth's field. Extension to dynamic interference cancellation based on simultaneous recordings from the reference sensors should be relatively straightforward since the relevant interference occurs at relatively low frequency (<150 Hz) and this approach can build on approaches developed for SQUID-based MEG systems (Taulu et al., 2014). The static fields reported by the on-sensor coils of the array of 18 OPMs contained within the scanner-cast were also recorded with and without field nulling before the start of the retinotopic mapping experiment while the subject viewed the centre of the screen. Taking the mean of the magnitude of these fields reveals a decrease from 14.9 ± 5.2 nT to 1.61 ± 0.43 nT after the field nulling was applied. The reduction in field is therefore less for the OPMs on the head than was found during the field-mapping. This is mainly due to the nature of the scanner-cast and the dimensions of the OPMs resulting in the subject having to sit with their head positioned forward of the reference array in the *x*-direction, to avoid hitting the reference sensors whilst moving during an experiment. A re-designed reference array could improve the nulling of fields over the scanner-cast. Nevertheless, the on-sensor coils can readily zero the residual fields at the sensors, and even with the significant head movements that were made in this experiment, none of the sensors went outside their operational range. We have shown in previous work (Boto et al., 2018) that small head movements cause the OPM sensors to saturate when field nulling using the bi-planar coil set is not applied.

The results shown in Fig. 7A indicate that the field measurements are highly correlated with the changes in head position, which is also evident from the large reduction in field values produced by regressing out the movement parameters that is shown in Fig. 7D. Linear regression with the motion parameters recorded by the OptiTrack, shown in Fig. 7, allows for artefacts associated with movement to be further reduced, prior to analysis either through averaging, performing a dipole fit or applying beamformer analysis. The standard deviation over time of the measured field averaged over all sensors during the experiment fell from 0.35 ± 0.05 nT to 0.034 ± 0.006 nT following the regression with the motion parameters and signals from the reference magnetometers. The artefact reduction method could be improved by implementing a non-linear solution as the regression weights that remove interference for one orientation will be different when the head is moved.

The bi-planar coils that we have used here were constructed by simple manual winding of the wires following a printed pattern and the wires were fixed in place using masking tape and wallpaper paste. 3D printing or printed circuit board etching techniques could potentially be used to streamline the process of coil construction and to increase the correspondence of the actual wire paths to the coil designs. In addition, our coils are affixed to MDF boards that are not completely flat and the positioning of the two boards carrying the bi-planar coil pairs is done manually, leading to the possibility of small errors in alignment and separation of the coil pairs. These effects, which could be eliminated by use of alternative materials and more accurate construction of the coil mountings, have not proved problematic in field nulling, but may underlie some of the small discrepancies between the measured and calculated coil characteristics that are evident in Table 2. Discrepancies may also have been caused by interactions between the coils and the high permeability mu-metal of the MSR which were not considered in the coil design process.

Further development of the field nulling technology will be required to allow full ambulatory motion during an experiment. To realise this aim, new designs could feature a larger region over which the homogeneous fields and field gradients are produced. For example, the size of the homogeneous region produced by the coils described here could be doubled simply by doubling the size of the planes. Additional coils producing higher order spatial variations of the field could also readily be produced. Alternatively, the aspect ratio of the coils could be altered to provide a “corridor” within which the field is made homogeneous. The coils could also be built directly into the walls of the MSR, but this would require careful consideration of the interactions between the coils and the mu-metal – similar interactions have previously been accounted for in gradient coil design for MRI (Moon et al., 1999). Any increase in the volume within which the OPMs remained operational would immediately allow for wider ranges of motion, and potentially make possible the implementation of experiments involving spatial navigation and direct social interaction between individuals.

### 4.2 OPM-MEG demonstration and future expansion

Our novel visual mapping paradigm, using head-direction to manipulate retinotopic stimulus location, demonstrates the new kinds of experimental paradigms possible with a wearable system. Previous functional imaging studies of the human visual system have primarily focused on paradigms where the head and gaze-direction remain fixed and stimuli move in retinotopic space (Sereno et al., 1995). Such paradigms, with minimal head motion, are clearly optimal for conventional neuroimaging. However, we know that the brain has to integrate information from multiple coordinate systems (head, gaze, body, hand-centred for example) in order to manoeuvre around and manipulate objects in the real world. These coordinate systems are also determined, and re-weighted based on multisensory input (Sereno and Huang, 2014). For example, the (predominantly) parietal body and face centred maps of personal space integrate visual and proprioceptive cues (Huang et al., 2012; Bernier and Grafton, 2010; Sereno and Huang, 2006) and recent evidence suggests that these space fields can even be modulated by gravitational cues (Bufacchi and Iannetti, 2016). The OPM technology, and its resilience to subject motion would allow one to non-invasively study millisecond resolved integration of these multiple sensory cues in both healthy participants and (for example) patients with spatial neglect.

The ability to allow large subject movements (>10 cm range of head translation and >10° range of head rotation), and have the sensors move with the head during a recording is a first for MEG, highlighting the potential for a step-change in functional neuroimaging based on magnetoencephalography. A completed system could be widely applied in the research environment; for example, the ability to make such large head movements would enable novel paradigms that are inaccessible to current scanning techniques. Additionally, the system could be used flexibly to assess development across the lifespan; providing invaluable information on the function of the human brain gathered from subjects from birth to old age – such measurements are challenging using cryogenic systems without specialised equipment. The system could also have significant clinical application, coupling reduced operating costs with the potential to provide improved assessment of the development of diseases, such as epilepsy and schizophrenia (Barkley and Baumgartner, 2003; Robson et al., 2016). The bi-planar field coils described here are crucial to allowing subject movement and the continued development of coil technology is needed to fully realise the potential of OPM-based MEG.

## Figures and Tables

**Fig. B1:**
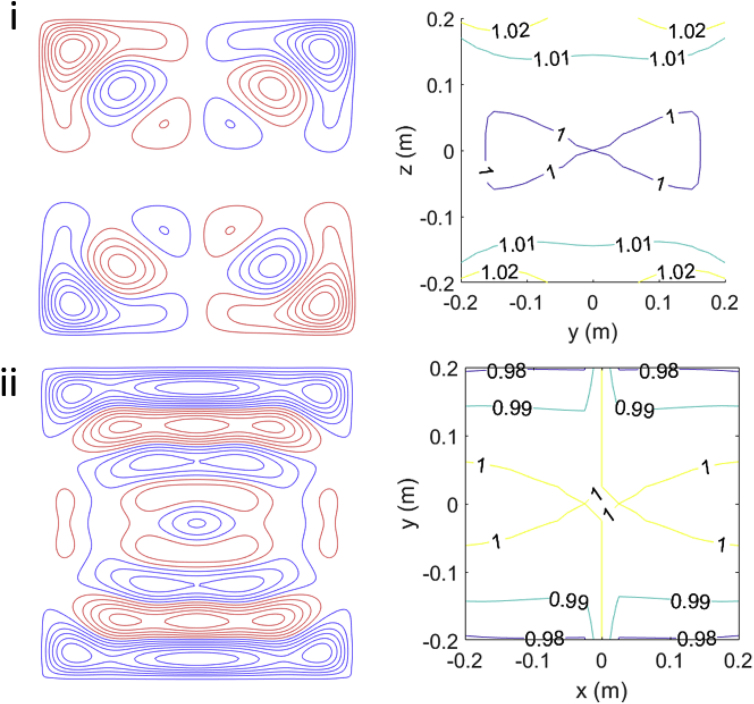
Wire paths and field contours for the i) $dB_{x}/dy$ and ii) $dB_{x}/dx$ coils. The contours are normalised to the field gradient in the centre of each coil pair and are shown for the region i) *x* = 0.2 cm, |*y*|, |*z*|<0.2 m ii) z = 0 cm, |x|, |y|<0.2 m. Red and blue denote wires carrying currents in opposing directions.

**Table B1:** Coil parameters calculated assuming copper wire of 0.56 mm diameter is used to wind the coils.

Coil	Length of wire (m)	Resistance of wire (Ω)	Gradient per unit current (nT/mA/m)	Inductance (μH)
$dB_{x}/dy$	97	6.83	0.41	154
$dB_{x}/dx$	173	12.1	0.40	562

## Appendix A Power dissipation term

MRI gradient coils are often designed with terms in the functional F (Eq. [8]) that act to minimise additional coil parameters alongside the deviation of the magnetic field from the target distribution. These include the power dissipation, inductance and torque (Carlson et al., 1992; Jin, 1998; Turner, 1993). As the coils designed here do not need to generate rapidly time-varying fields and are not operated inside a large magnetic field, inductance minimisation and torque balancing are unnecessary and only the power term needs to be included in the functional.

Considering a current distribution $J=J_{x}\left(x,y\right)\hat{x}+J_{y}\left(x,y\right)\hat{y}$ confined to an (x−y) plane of area L×L, thickness t and resistivity ρ, the power dissipation is given by

(S1) $$P=I^{2}R=\frac{\rho }{t}\int_{-L}^{L}dx\int_{-L}^{L}dy\;\left(J_{x}^{2}+J_{y}^{2}\right)\;.$$

Then by applying Parseval's relation and recalling the definition of the stream function ($\nabla \text{S}\times \hat{z}=J$) the power dissipation can be expressed as

(S2) $$P=\text{Ω}\int_{-L}^{L}dk_{x}\int_{-L}^{L}dk_{y}\;k_{r}^{2}\;{\left|\tilde{S}\right|}^{2}$$

where Ω is introduced to represent the combined effect of all constants. This term is then weighted by a tuneable term ω before inclusion in the functional. Since $\;S=\sum_{j=1}^{N\times M}\lambda_{j}S_{j},$ Eq. [S2] can be rewritten as

(S3) $$P=\text{Ω}\int_{-L}^{L}dk_{x}\int_{-L}^{L}dk_{y}\;k_{r}^{2}\;{\left|\sum_{j}{\tilde{S}}_{j}\lambda_{j}\right|}^{2}$$

Since the power is minimised by reducing local large excursions in the amplitude of the stream function, higher values of ω result in simpler wire paths, but this is associated with a loss of field or gradient homogeneity. Lower values of ω result in more homogenous fields formed from more complex wire paths. In designing each coil the value of Ω was adjusted to the minimum value that produced deviation of less than 5% of the field or gradient from uniformity within the 40 × 40 × 40 cm^3^ target region, thus yielding buildable coils that satisfied the homogeneity requirements.

## Appendix B Additional coils

As described in Section 2.1, in order to fully characterise the field and first-order field gradients 8 distinct coils are required. Here we have provided designs and results for 6 of these coils. The designs of the two remaining coils which produce gradients of the form $dB_{x}/dy$ ($=dB_{y}/dx$) and $dB_{x}/dx$ ($=-dB_{y}/dy-dB_{z}/dz$) are now described here.

The $dB_{x}/dy$ coil was designed using the method outlined in Section 2.1 using 16 harmonics (N = M = 4) which are anti-symmetric in *x*, *y* and *z*. The field was evaluated at I = 320 target points. As before the region over which a homogeneous field gradient (within ±5%) is produced is 40 × 40 × 40 cm^3^ in extent. The wire paths and field contours normalised relative to the field gradient at the centre of each coil pair are shown in Figure A1 (i).

Since the symmetry of the bi-planar coil arrangement needed to produce the $dB_{x}/dx$ field gradient (symmetric in *x* and *y* and anti-symmetric in *z*) is the same as that of the coil arrangement that produces a field gradient $dB_{z}/dz$ ($=-2dB_{x}/dx=-2dB_{y}/dy$), steps had to be taken to force the field to follow the right spatial form. This meant breaking the 4-fold symmetry of the stream function for rotation about the *z*-axis which was inherent in the design of the $dB_{z}/dz$ coil. This was done by removing the first component of the stream function in the x dimension in Eq. [4]. Thus the stream function components with n=2,3,4,5 andm=1,2,3,4,5 were included. The field was evaluated at I = 320 target points. The wire paths and normalised field gradient contours are shown in Figure B1 (ii). Table B1 shows the simulated coil parameters assuming the coils are wound using 0.56 mm diameter copper wire.

Addition of these two coils to the 6 coils that have already been constructed would clearly further improve the field nulling system, but would require inclusion of additional reference sensors in the reference array. These would be needed to allow separate characterisation of.

$dB_{x}/dy$ or $dB_{y}/dx$, and $dB_{x}/dx$ or $dB_{y}/dy$. This would require a modified construction in which two reference sensors are separated in the *x* or *y*-direction. Further work is needed to establish whether the benefits of including these two additional coils, given the spatial form of the residual field in our MSR, justify the effort that would be required to construct them and a new reference array.
